# Psychosocial and adaptive behavior functioning in children following pediatric intensive care unit hospitalization: prospective cohort study

**DOI:** 10.1186/s12887-025-06156-9

**Published:** 2025-09-18

**Authors:** Reham I. Abdelmageed, Azza M. Youssef, Sondos M. Magdy, Ahmed N. Nasr, Asmaa W. Abdelaziz

**Affiliations:** Pediatrics Department, Faculty of Medicine, Ain Shams University, Cairo, Egypt

**Keywords:** Children, PICU, Psychosocial health, Adaptive behavior, Functioning

## Abstract

**Background:**

With increasing survival rates following pediatric intensive care unit (PICU) hospitalization, assessing functional outcomes is imperative. We compared children’s psychosocial and adaptive functioning six weeks and six months after discharge from the PICU with children hospitalized in a general ward.

**Methods:**

This prospective observational cohort study followed 100 children aged 2 to 11 years after PICU and ward discharge (control group) for six months. The two groups were compared for psychosocial and adaptive functioning using the Behavioral Assessment System for Children, Third Edition (BASC-3) parent report scale. Parents also completed the Parenting Stress Index-Short Form scale to assess parenting stress. Multivariate regression analysis adjusted for demographics, PICU-related factors, and parental stress was used to investigate the potential variables associated with children’s psychosocial and adaptive functioning six weeks and six months after PICU discharge.

**Results:**

Our findings indicated that children experienced greater psychosocial problems and worse adaptive functioning up to six months after PICU discharge than children after ward discharge, *P* < 0.05. The number of intervention procedures and parental stress were common factors associated with impaired psychosocial and adaptive functioning post-PICU discharge. Six months after PICU discharge, younger age and length of stay were associated with psychosocial problems, while internalizing and externalizing problems were associated with poorer adaptive functioning, *P* < 0.05.

**Conclusion:**

Given the impact of PICU experience on children’s psychosocial and adaptive outcomes, it is crucial to implement psychological assessments with a follow-up plan extended longitudinally after PICU discharge. This study underscores the need to integrate adaptive behavior assessment in children’s psychological evaluation to identify significant deficits, develop relevant behavioral management plans, and monitor their progress.

## Introduction

The pediatric intensive care unit (PICU) is a challenging environment, characterized by enduring stress and anxiety in children [1, 2]. Children in the PICU experience numerous painful and stressful procedures [3], surrounded by chaotic and noisy environments, separated from their families [4], and cared for by multiple strangers, which can lead to physical and behavioral responses [4].

Research has described a wide range of psychological problems that children may face after being in the PICU, including posttraumatic stress disorder (PTSD) [5], psychosocial problems [6], attention deficit [7], cognitive dysfunction [8], and developmental delay [9].These psychological difficulties significantly burden children after PICU discharge and may persist for many years [10, 11].

Adaptive functioning refers to the skills individuals need to effectively communicate, socialize, and be independent in their daily lives [12]. Adaptive skills include functional communication, daily living skills, leadership, and social skills, which help children manage daily life changes and the environment [13]. Impairments in adaptive behavior may prevent an individual from achieving age-appropriate standards of independence and community engagement [14].

Few studies have investigated adaptive functioning outcomes of children admitted to the PICU [15–17]. Biagas et al. [15] found no differences in one-year adaptive behavior outcomes on the Vineland Adaptive Behavior Scale (VABS-2) in critically ill children on two types of glycemic control. A randomized clinical study evaluated the effectiveness of therapeutic hypothermia compared to normothermia in improving neurobehavioral outcomes in comatose children who survived cardiac arrest. The study found no significant differences between the two groups regarding survival with good functional outcomes measured by the VABS-2 after one year. Ebrahim et al. [17] examined adaptive behavior in a sample (*n* = 65) of children and adolescents aged one month to 18 years who were urgently admitted to the ICU. The parents reported low adaptive functioning scores on the (VABS-2). The study revealed that adaptive behavior was associated with the intensity of resuscitation, the severity of illness, and the reason for admission to the ICU. However, the follow-up period was one month, which was considered relatively short to assess long-term adaptive functioning.

Several studies have reported a range of psychological problems in children after PICU admission [18]. However, little is known about their adaptive functioning following this experience. Thus, this study aimed to investigate children’s psychosocial and adaptive functioning six weeks and six months after PICU discharge and compare it to children admitted to a general ward. We also examined the factors related to children’s psychosocial and adaptive functioning six weeks and six months after PICU hospitalization. We hypothesized that children would have more psychosocial problems and lower adaptive functioning six weeks after PICU discharge than six weeks after general ward discharge. Furthermore, psychosocial issues and lower adaptive functioning would persist six months post-PICU discharge compared with those who were discharged from a general ward. We also hypothesized that children’s psychosocial and adaptive functioning post-PICU discharge would be related to child age, PICU-related factors (length of stay, intervention procedures), and family-related factors (parenting stress).

## Patients and methods

### Study population

This prospective observational cohort study included 50 children recruited from the pediatric intensive care unit (PICU) (study group), Pediatric Hospital, Faculty of Medicine, Ain Shams University. Another 50 children admitted to the same hospital’s general pediatric wards were also selected to form a control group. The study was conducted between April 2021 and December 2022. Eligible children had been in the PICU or ward for at least 48 h and were expected to be discharged. The children and their caregivers had a good comprehension of Arabic, and their ages ranged from 2 to 11 years. Children who suffered from neurological impairment or psychiatric disorders were excluded from the study. Children from the PICU were matched with children in a ward group based on age and whether their illness was acute or chronic. Moreover, we excluded children with a history of previous PICU admission from the general ward group.

Ethics Committee No. FMASU MS 678, the ethics committee of Ain Shams University Hospitals, granted ethical permission for the study, which followed the 1975 Helsinki Declaration.

### Procedure

We approached 132 parents of eligible children to describe the study, address questions, and obtain parental consent. Of these, 9 refused participation, and 23 did not reply during the follow-up period. The final sample consisted of 100 children, with 50 in each group.

The first data collection point was six weeks after discharge from the PICU or general ward. Two questionnaires were mailed to parents to complete at home: the Behavior Assessment System for Children (BASC − 3) [13] to indicate their child’s psychosocial and adaptive behavior characteristics and the Parenting Stress Index (PSI-4-SF) [19] to assess parenting stress.

After sending out the questionnaires, we contacted parents by phone to follow up and encouraged them to complete and return the forms by email. The phone calls included questions about the child’s condition and any concerns the parents may have had. We provided the option of having a house courier pick up the forms. Data collection continued for six months post-discharge, using the two questionnaires and reminder telephone calls to ensure complete follow-up.

Demographic data were collected from the parents, and detailed medical history was collected from medical records, including (medical diagnosis, number of previous hospital or PICU admissions, length of stay, Pediatric Risk of Mortality-III (PRISM) score, and Therapeutic Intervention Scoring System (TISS-28). We assessed socioeconomic standards (SES) using an Arabic-validated Socioeconomic Status Scale designed for health research [20].

### Measures

#### Pediatric risk of mortality-III (PRISM) (21)

The PRISM score is a commonly used index measure of illness severity based on 17 physiological parameters and their ranges. The PRISM scoring system assigns scores ranging from 0 to 75, reflecting the severity of a patient’s condition and prognosis. The mean score was calculated based on scores obtained within 24 h of admission.

#### Therapeutic intervention scoring system (TISS-28) (22)

The TISS-28 is a commonly used instrument for documenting the frequency and types of intervention procedures each child underwent during hospitalization. It indicates illness severity based on the number of medical interventions needed.

#### Behavioral assessment system for children, third edition (basc − 3) parent report

The BASC-3 is a comprehensive set of rating scales used to assess psychosocial health and adaptive functioning in children and adolescents aged 2–21 years based on parent and teacher ratings [13].

In this study, we utilized the Parent Rating Scale (PRS-P), a preschool version (for children aged 2–5 years) and a child version (for children aged 6–11 years). The preschool version consisted of 139 items, while the child version consisted of 175 items. The items assess psychosocial and adaptive functioning across various clinical subscales, contributing to four composite score indices. Internalizing problems composite (depression, anxiety, and somatization); externalizing problems composite (aggression, hyperactivity, and conduct problems); and the behavioral symptoms index composite (BSI) (attention problems, atypicality, and withdrawal). The adaptive skills composite analyzed adaptive functioning based on five domains: adaptability (adjusting abilities to changes in the surrounding circumstances), social skills (effectively interacting with others in various contexts), leadership (capability for teamwork and accomplishing academic and community objectives), activities of daily living (fulfilling basic and daily chores safely and acceptably), and functional communication (explaining and communicating thoughts to others in an understandable way) [13].

Parents completed scales using four-point Likert responses ranging from 0 (never) to 3 (almost always). Except for the adaptive skills composite, T scores between 60 and 69 are classified as at-risk, while T scores of 70 or higher are classified as clinically significant. For the adaptive skills composite, T scores between 31 and 40 are classified as at-risk, and scores of 30 or lower are classified as clinically significant.

The BASC-3 PRS-P has a broad spectrum of psychometric properties, ranging from acceptable to excellent. This is evidenced by Cronbach’s alpha coefficient values that range from 0.70 to 0.98 [13]. The license agreement and license for use and translation of the Arabic version were granted by Pearson Clinical Assessment. In this study, Cronbach’s alpha coefficients ranged from 0.79 to 0.84 for the preschool form (2–5 years) and from 0.83 to 0.88 for the school-age form (6–11 years).

#### Parenting stress index-fourth edition-short form (PSI-4-SF)

The PSI-SF is a self-report inventory consisting of 36 items that are rated on a 5-point Likert scale [19]. The scale comprises three subscales, each containing 12 items that quantitatively evaluate various aspects of parenting stress: parental distress (PD), parent-child dysfunctional interaction (P-CDI), and difficult child (DC). The total score (PSI-TS) is the sum of the scores from the three domains. Scores in the 85th percentile or higher indicate clinically significant stress levels.

The publisher, Psychological Assessment Resources, Inc., provided the Arabic-translated form in this study after obtaining a signed license agreement. The Arabic version of the stress scale was tested on the population of Arabic parents, and the Cronbach’s alpha coefficient for the total scale was 0.9, demonstrating adequate psychometric properties [23]. In this study, the Cronbach’s alpha coefficient for the total scale was 0.85.

### Statistical analysis

Using the PASS 11 program to calculate sample size, setting power at 80%, and alpha error at 5%, after reviewing results from an earlier study [1], which revealed that 21% of PICU-discharged children developed psychological problems compared to none of the children admitted to the general hospital ward. Thus, a sample size of at least 100 children (50 per group) is required.

The data provided were analyzed using SPSS© Statistics 25.0. The quantitative data are presented as the mean and standard deviation, whereas the qualitative data are expressed as numbers and percentages. Student’s t-tests, chi-square tests, and Fisher’s exact tests were used to compare children in the PICU with children in the general ward. We conducted univariate regression analyses on the four composites of the BASC-3-P to screen for potential variables influencing psychosocial responses and adaptive functioning at six weeks and six months post-discharge in the PICU group. The variables included demographic characteristics (age, sex, socioeconomic status), disease variables (total number of readmissions and hospital visits following discharge, severity of illness, number of intervention procedures, length of stay), and total parenting stress. Also, we added internalizing and externalizing scores in the regression analysis of adaptive functioning to explore the relationship between psychosocial problems and adaptive functioning. We included the variables with a significant association with four composites of the BASC-3 in the multivariate regression analysis.

## Results

Demographics and clinical characteristics are displayed in Table 1. The pediatric intensive care unit (PICU) and ward groups did not significantly differ in age (*p* = 0.321), sex (*p* = 0.841), residency (*p* = 0.838), or socioeconomic status (*p* = 0.736). Also, in both groups, mothers constituted the most participating parents (*p* = 0.825). There were significant differences between the two groups in the number of previous hospital admissions (*p* = 0.033), length of stay (*p* = 0.003), severity of illness (*p* < 0.001), and intervention procedures scores (*p* < 0.001). Moreover, the total number of children readmitted and hospital visits six weeks post-discharge was greater in the PICU group (*p* = 0.003); however, this difference was not observed at six months (*p* = 0.806).

**Table 1:** Demographics and clinical characteristics of the ward and pediatric intensive care unit groups

		Ward (*N* = 50)	PICU (*N* = 50)	*p*-Value
		Mean ± SD *N* (%)	Mean ± SD *N* (%)	
Age of the child (year) Range (2–11 years)		6.16 ± 3.08	6.78 ± 3.14	0.321
Gender	Male	26 (52%)	25 (50%)	0.841
	Female	24 (48%)	25 (50%)	
Residency	Urban	19 (38%)	20 (40%)	0.838
	Rural	31 (62%)	30 (60%)	
Age of mother/father		32.02 ± 4.53	32.74 ± 3.97	0.421
Type of respondents	Father	14 (28%)	15 (30%)	0.825
	Mothers	36 (72%)	35 (70%)	
Socioeconomic Status (SES)		29.74 ± 8.06	30.26 ± 7.32	0.736
SES	Low	7 (14%)	6 (12%)	0.288
	Medium	33 (66%)	27 (54%)	
	High	10 (20%)	17 (34%)	
Diagnosis category	GIT	7 (14%)	5 (10%)	0.745
	Respiratory	21 (42%)	24 (48%)	
	Surgery	13 (26%)	8 (16%)	
	Trauma	6 (12%)	7 (14%)	
	Oncology	1 (2%)	3 (6%)	
	Cardiac	0 (0%)	1 (2%)	
	Others	2 (4%)	2 (4%)	
Number of previous hospital admissions	0	36(72%)	33 (66%)	0.274
	1	13 (26%)	10 (20%)	
	2	1 (2%)	6 (12%)	
	4	0 (0%)	1 (2%)	
Number of Previous ICU Admission	0	50 (100%)	47 (94%)	0.242
	1	0 (0%)	3 (6%)	
Total number of readmissions and hospital visits following discharge	6 weeks	6 (12%)	19(38%)	0.003**
	6 months	1 (2%)	8(16%)	0.806
Length of stay (days)		5.58 ± 3.06	8.46 ± 5.8	0.003**
Previous hospital admission of other siblings	No	43 (86%)	45 (90%)	0.538
	Yes	7 (14%)	5 (10%)	
PRISM 3		3.02 ± 1.19	9.5 ± 5.93	< 0.001**
TISS-28		6.7 ± 2.5	14.98 ± 8.61	< 0.001**

*PRISM* Pediatric Risk of Mortality, *TISS* Therapeutic intervention scoring system, *N* number, *SD* standard deviation

** *P* < 0.01 level

## Children’s psychosocial functioning

Table 2 shows the mean scores and percentages in the at-risk/clinically significant ranges on the BASC-3 clinical subscales and composites at six weeks and six months post-discharge.

**Table 2:** Psychosocial function: comparing ward and PICU groups at 6 weeks and 6 months

Variable	Ward (*N* = 50)	ICU (*N* = 50)	Student t-test		Ward (*N* = 50)	ICU (*N* = 50)	Fisher’s Exact test
	Mean ± SD	Mean ± SD	T	*p*-Value	AR/CS (%)	AR/CS (%)	*p*-Value
Clinical subscales/Composite							
Externalizing problems composite 6 Weeks	54.75 ± 15.14	73.43 ± 20.67	−3.524	0.001**	8	34	0.003**
Hyperactivity 6 Weeks	51.46 ± 12.53	68.65 ± 19.21	−3.617	0.001**	6	36	0.003**
Aggression 6 Weeks	51.13 ± 14.03	69.57 ± 21.72	−3.441	0.001**	10	32	0.013*
Conduct problems 6 Weeks	58.46 ± 22.7	71.87 ± 22.79	−2.021	0.049*	14	32	0.083
Externalizing problems composite 6 Months	50.04 ± 16.74	64.91 ± 23.1	−2.518	0.016*	4	30	0.002**
Hyperactivity 6 Months	54.04 ± 12.48	66.57 ± 17.52	−2.832	0.007**	4	30	0.005**
Aggression 6 Months	54.25 ± 10.79	64.3 ± 19.71	−2.156	0.038*	6	28	0.025*
Conduct problems 6 Months	48 ± 22.74	68.04 ± 26.79	−2.002	0.048*	8	26	0.056
Internalizing problems composite 6 Weeks	50.06 ± 20.59	68.56 ± 20.63	−4.488	< 0.001**	8	36	0.002**
Anxiety 6 Weeks	58.52 ± 17.44	69.7 ± 21.48	−2.858	0.005**	12	38	0.014*
Depression 6 Weeks	47.18 ± 21.23	65.14 ± 21.5	−4.203	< 0.001**	6	24	0.024*
Somatization 6 Weeks	44.08 ± 18.58	65.46 ± 23.29	−5.074	< 0.001**	4	30	0.001**
Internalizing problems composite 6 Months	44.4 ± 21.31	64.38 ± 21.88	−4.625	< 0.001**	6	32	0.006**
Anxiety 6 Months	52.76 ± 18.2	66.02 ± 22.53	−3.237	0.002**	10	26	0.031*
Depression 6 Months	41.1 ± 19.66	63.2 ± 23.16	−5.143	< 0.001**	4	26	0.004**
Somatization 6 Months	40.18 ± 19	58.84 ± 26.66	−4.030	< 0.001**	4	24	0.017*
Behavioral symptoms Index 6 Weeks	47.2 ± 5.44	51.54 ± 8.49	−3.045	0.003**	6	28	0.032*
Attention problems 6 Weeks	50.64 ± 5.42	56.1 ± 10.78	−3.199	0.002**	10	32	0.043*
Atypicality 6 Weeks	44.94 ± 2.87	47.06 ± 7.65	−1.836	0.071	0	6	0.117
Withdrawal 6 Weeks	48 ± 7.59	50.34 ± 7.79	−1.521	0.131	8	16	0.556
Behavioral symptoms Index 6 Months	46.04 ± 5.32	49.88 ± 8.76	−2.649	0.01*	4	24	0.038*
Attention problems 6 Months	49.38 ± 6.29	54.52 ± 10.05	−3.066	0.003**	6	26	0.041*
Atypicality 6 Months	44.76 ± 2.41	46.96 ± 7.64	−1.941	0.057	0	2	1.00
Withdrawal 6 Months	46.56 ± 5.3	48.84 ± 7	−1.836	0.069	2	12	0.193

Behavioral Assessment System for Children, Third Edition (BASC − 3) Parent Report; was used to measure symptoms

*P < 0.05 level

** *P* < 0.01 level, AR/CS, At-risk/Clinically significant

According to the parent measures, at six weeks post-discharge, children in the PICU group scored significantly higher on all clinical subscales except the atypicality and withdrawal subscales (*p* = 0.071 and 0.131, respectively). Further, the significance of the Behavioral Symptoms Index subscales was on attention subscale scores only (*p* < 0.002). They also showed more significant impairment that persisted at six months post-discharge, compared to the ward group.

Furthermore, children in the PICU group showed significantly greater psychosocial problems at the composite level than those in the ward group. This difference was observed at six weeks and six months post-discharge, as indicated by elevated mean scores for externalizing problems (6wks *p* = 0.001; 6ms *p* = 0.016), internalizing problems (*p* < 0.001), and the behavioral symptoms index (6wks *p* = 0.003; 6ms *p* = 0.01).

Within the clinical subscales, children in the PICU group displayed a significantly greater percentage of scores falling within the risk/clinically significant range at six weeks post-discharge. These subscales include hyperactivity (36% vs. 6%, *p* = 0.003), aggression (32% vs. 10%, *p* = 0.013), anxiety (38% vs. 12%, *p* = 0.014), depression (24% vs. 6%, *p* = 0.024), somatization (30% vs. 4%,*p* = 0.001), and attention problems (32% vs. 10%, *p* = 0.043)compared to the ward group. This significant difference in the percentage of children in the PICU group classified as at risk/clinically significant range continued six months post-discharge. Similarly, a substantial percentage of children in the PICU group fell within the at-risk/clinically significant range at both six weeks and six months post-discharge on the externalizing problem composite (6wks 34% vs. 8%, *p* = 0.003; 6ms 30% vs. 4%, *p* = 0.002), internalizing problems composite (6wks 36% vs. 8%, *p* = 0.002; 6ms 32% vs. 6%, *p* = 0.006), and behavioral symptoms index composite (6wks 28% vs. 6%, *p* = 0.032; 6ms 24% vs. 4%, *p* = 0.038).

### Children’s adaptive functioning

Table 3 shows the mean scores and percentages in the at-risk/clinically significant ranges on the BASC-3 parent reports for the adaptive subscale and composite at six weeks and six months post-discharge.

**Table 3:** Adaptive function: comparing ward and PICU groups at 6 weeks and 6 months

Variable	Ward (*N* = 50)	ICU (*N* = 50)	Student t-test		Ward (*N* = 50)	ICU (*N* = 50)	Fisher’s Exact test
	Mean ± SD	Mean ± SD	T	*p*-Value	AR/CS (%)	AR/CS (%)	*p*-Value
Adaptive Skills Composite 6 Weeks	44 ± 14.7	37.3 ± 16.62	2.135	0.035*	12	32	0.034*
Adaptability 6 Weeks	43.83 ± 14.56	30.57 ± 16.05	3.059	0.004**	6	28	0.002**
Social Skills 6 Weeks	56.68 ± 22.5	41.54 ± 19.4	−3.603	< 0.001**	12	30	0.001**
Leadership 6 Weeks	48.65 ± 4.15	41.32 ± 5.24	3.217	0.002**	5	14	0.021*
Activities of Daily Living 6 Weeks	40.3 ± 16.35	39.44 ± 18.73	0.245	0.807	6	10	0.487
Functional communication 6 Weeks	46.3 ± 14.7	41.92 ± 16.95	0.120	0.705	2	10	0.237
Adaptive Skills Composite 6 Months	46.14 ± 20.73	35.78 ± 21.12	2.476	0.015*	8	30	0.023*
Adaptability 6 Months	44.96 ± 16.86	31 ± 15.78	3.048	0.004**	4	26	0.002**
Social skills 6 Months	51.36 ± 24.95	37.2 ± 19.4	−3.168	0.002**	8	22	0.018*
Leadership 6 Months	49.13 ± 4.9	44.61 ± 5.24	3.156	0.003**	3	10	0.037*
Activities of Daily Living 6 Months	46.86 ± 14.79	42.78 ± 19.01	1.198	0.234	2	8	0.204
Functional communication 6 Months	47.42 ± 16.01	42.48 ± 14.44	−0.308	0.759	0	4	0.794

Behavioral Assessment System for Children, Third Edition (BASC − 3) Parent Report; was used to measure symptoms

*P < 0.05 level

** *P* < 0.01 level, AR/CS, At-risk/Clinically significant

Compared with those in the ward group, the children in the PICU group scored significantly lower (i.e., worse functioning) at six weeks and six months post-discharge on adaptability (*p =* 0.004), social skills (6wks *p* < 0.001; 6ms *p* = 0.002), and leadership (6wks *p* = 0.002; 6ms *p* = 0.003). Overall, during the follow-up periods, children in the PICU group showed lower adaptive functioning, as indicated by lower mean scores on the adaptive skills composite, than those in the ward group (6wks *p* = 0.034; 6ms *p* = 0.023).

At six weeks post-discharge, 32% of the children in the PICU group were in the at-risk/clinically significant range on the adaptive skills composite compared to 12% in the ward group (*p* = 0.034). This considerable difference continued for six months post-discharge (30% vs. 8%, *p* = 0.023).

#### Parent stress level

Overall, total parenting stress using PSI-4-SF showed that parents of children in the PICU group scored significantly higher on PSI-TS compared to parents of children in the ward group at six weeks (57.74 ± 9.52 vs. 40.71 ± 5.48; *P =* 0.001) and six months post-discharge (54.48 ± 9.34 vs.38.58 ± 5.93; *p* = 0.014). Furthermore, parents of children in the PICU group displayed a significantly greater percentage of scores falling within the clinically significant range compared to parents of children in the ward group at six weeks (43.48% vs. 8.33%; *P =* 0.008) and six months post-discharge (30.43%vs. 4.35%; *P =* 0.024).

#### Regression models analysis for factors associated with psychosocial and adaptive functioning in children admitted to the PICU

We performed univariate and multivariate analyses to determine factors associated with psychosocial and adaptive functioning at six weeks and six months post-discharge in the PICU group.

At six weeks post-discharge, disease variables (severity of illness, number of intervention procedures, length of stay) and total parenting stress were significantly associated with greater psychosocial problems. These factors were associated with higher scores for the externalizing problem composite (F = 4.85, *p* < 0.05, adjusted *R*^***2***^ = 0.33), internalizing problems composite (F = 4.47, *p* < 0.05, adjusted *R*^***2***^ = 0.31), and behavioral symptoms index composite (F = 5.66, *p* < 0.05, adjusted *R*^***2***^ = 0.36) (Table 4).

**Table 4:** Regression analyses of variables of psychosocial composite scores in the PICU group (*n* = 50) at 6 weeks and 6 months

Variable	Externalizing problems composite 6 Weeks	Externalizing problems composite 6 Months	Internalizing problems composite 6 Weeks	Internalizing problems composite 6 Months	Behavioral symptoms Index 6 Weeks	Behavioral symptoms Index 6 months
	B	B	B	B	B	B
Univariate						
Total number of readmissions and hospital visits following discharge	1.153	1.192	1.141	1.122	1.098	0.413
PRISM 3	1.546*	1.289*	1.437*	1.422*	1.565*	1.296*
TISS-28	1.787**	1.369*	1.676**	1.262*	1.596*	1.297*
Age of the child (years)	−1.106**	−1.459**	−0.985**	−0.897**	−0.423*	−0.412*
Male Sex	−1.944	−2.151	−0.924	−0.970	−2.447	−2.569
Urban Residency	−1.633	−1.022	1.072	1.070	−0.084	−0.088
Socio-economic status	−1.238	−0.165	−0.044	−0.046	−0.083	−0.087
Length of say in ICU (days)	1.856**	1.691*	1.589*	1.668*	1.620*	1.701*
PSI -TS score 6wks	1.784**		2.004**		1.731**	
PSI -TS score 6ms		1.470*		2.105**		1.818**
Multivariate analysis						
PRISM 3	1.399*	0.553	1.337*	0.491	1.465*	0.596
TISS − 28	1.424*	0.445	1.325*	0.994	1.237*	0.369
Age of the child (years)	−0.446*	−0.459*	−0.555*	−0.583*	0.286	0.300
Length of say in ICU (days)	1.358*	1.381*	1.325*	1.318*	1.298*	0.761
PSI -TS score 6wks	1.396*		1.424*		1.331*	
PSI -TS score 6ms		1.280*		1.445*		0.298

Behavioral Assessment System for Children, Third Edition (BASC − 3) Parent Report; was used to measure symptoms

*B* unstandardized regression coefficient,*PRISM*Pediatric Risk of Mortality, *TISS* Therapeutic intervention scoring system PSI-TS Parenting Stress Index total stress score

*P < 0.05 level

**P < 0.01 level

At six months post-discharge, only length of stay and total parenting stress were significantly associated with externalizing problem composite (F = 4.36 *p* < 0.05, adjusted *R*^***2***^ = 0.30) and internalizing problems composite (F = 4.90, *p* < 0.05, adjusted *R*^***2***^ = 0.33) (Table 4). Moreover, younger children tended to have higher scores on the internalizing and externalizing problems composite throughout the follow-up period (Table 4).

Table 5 presents the variables related to adaptive functioning. Six weeks post-discharge, the number of intervention procedures, child age, internalizing and externalizing problems, and total parenting stress were significantly associated with adaptive skills composite scores (F = 3.81, *p* < 0.05, adjusted *R*^***2***^ = 0.28). Moreover, six months post-discharge, the age, total parenting stress, and internalizing and externalizing problems were significantly associated with adaptive skills composite scores (F = 2.84, *p* < 0.05, adjusted R2 = 0.22). Older age, lower levels of parenting stress, and fewer internalizing and externalizing problems were significantly associated with greater adaptive functioning.

**Table 5:** Regression analyses of variables of adaptive composite scores in the PICU group (*n* = 50) at six weeks and six months

Variable		Adaptive Skills Composite 6 Weeks			Adaptive Skills Composite 6 months		
		Β	t-value	*P*-value	Β	t-value	*P*-value
Univariate analysis							
Total number of readmissions and hospital visits following discharge		−0.201	−1.390	0.198	−0.249	−1.762	0.081
PRISM 3		0.697	0.743	0.116	0.761	0.996	0.445
TISS − 28		−0.946	−2.892	0.015*	−0.882	−2.672	0.013*
Age of the child (years)		0.754	3.584	0.003**	0.855	2.856	0.008**
Male Sex		0.949	0.963	0.380	0.498	0.496	0.670
Urban Residency		−1.948	−1.975	0.066	−1.686	−1.679	0.119
Socio-economic status		−0.117	−1.908	0.076	−0.025	−0.406	0.735
Length of say in ICU (days)		−0.042	−0.324	0.796	0.033	0.243	0.859
Externalizing problems composite	6 wks	−1.599	2.789	0.016*			
	6 ms				−1.789	2.941	0.012*
Internalizing problems composite	6 wks	−1.887	3.395	0.005**			
	6 ms				−1.672	2.899	0.007**
PSI -TS score	6 wks	−1.769	2.864	0.003**			
	6 ms				−1.623	2.875	0.008**
Multivariate analysis							
TISS − 28		−0.725	−2.327	0.026*	−0.072	−0.643	0.569
Age of the child (years)		0.564	2.522	0.015*	0.458	2.124	0.019*
Externalizing problems composite	6 wks	−1.389	2.579	0.027*			
	6 ms				−1.489	2.631	0.036*
Internalizing problems composite	6 wks	−1.237	2.355	0.045*			
	6 ms				−1.455	2.419	0.032*
PSI -TS score	6 wks	−1.141	1.727	0.033*			
	6 ms				−1.237	2.355	0.045*

*B* unstandardized regression coefficient, *PRISM* Pediatric Risk of Mortality, *TISS* Therapeutic intervention scoring system *PSI-TS* Parenting Stress Index total stress score

Behavioral Assessment System for Children, Third Edition (BASC − 3) Parent Report, was used to measure symptoms

*P < 0.05 level

** *P* < 0.01 level

## Discussion

This study examined the parent-reported psychosocial and adaptive functioning of preschool-aged and school-aged children admitted to the PICU. Overall, in this clinical sample, children discharged from the PICU experienced various psychosocial difficulties that warrant clinical consideration. Specifically, post-PICU discharge children had significantly more symptoms of hyperactivity, aggression, conduct problems, anxiety, depression, somatization, and attention problems than did post-ward discharge children at 6 weeks and 6 months post-discharge.

PICU admission entails a cascade of stressors [24], subjecting children to multiple types of trauma and negative experiences and overwhelming their limited psychological reserves to cope with adversity. Our findings indicated that more than 30% of children exhibited scores within the risk/clinically significant range for internalizing and externalizing problem composites six weeks after PICU discharge. Earlier research suggested that stressful events significantly impact the continuity and coexistence of externalizing and internalizing problems from early childhood to adolescence [25]. The authors suggested that externalizing problems could lead to the development of internalizing problems and that internalizing issues could influence the manifestation of externalizing problems. Stressful events were identified as factors influencing these relationships [25]. Several possible explanations for this reciprocal relationship include shared risk factors and one disorder increasing the risk for the other [26]. Our findings align with previous studies on psychosocial outcomes post-PICU discharge. These studies reported higher scores on the Strengths and Difficulties Questionnaire [6] and Child Behavior Checklist [27], indicating psychosocial problems.

Our results revealed the persistence of psychosocial problems for up to six months post-PICU discharge. This finding is similar to that reported in previous literature on the continuation of emotional and behavioral problems among children for years after being admitted to the PICU [6, 28]. These findings supported the stress generation theory, which suggests that individuals who experience elevated psychological problems are more likely to experience self-dependent-generated stressors [29]. The occurrence of greater dependent stress may have a significant influence on maintaining or worsening symptoms of mental illness, ultimately leading to a vicious cycle of increasing and chronic psychological problems [30].

This study captured adaptive functioning among children following discharge from the PICU for up to 6 months. Our data indicate that children experienced worse adaptive function up to six months after PICU discharge than children after ward discharge. Furthermore, approximately 30% of PICU survivors are at risk or have clinically significant scores on the general adaptive skills composite. Our current analysis revealed that post-PICU discharge children had problems with emotional self-regulation, particularly when faced with unexpected situations and during transitions between tasks (BASC-3 adaptability subscale). They also had difficulties engaging in social interactions (BASC-3 social skills subscale), making decisions, and achieving academic or community goals (BASC-3 leadership subscale).

Traumatic stress can significantly impair cognitive abilities, including mental flexibility, emotional self-regulation, adaptive and goal-focused behaviors, problem-solving, and engagement in school and relationships, which are essential for acquiring life-care skills [31, 32]. These executive function challenges often lead to significant mood and energy changes, which affect concentration, decision-making, social interactions, and the ability to adapt swiftly to various social demands [33].

Analyzing the potential factors that may influence psychosocial and adaptive functioning in children post-PICU discharge reveals an interesting perspective.

First, our results revealed that young age is associated with more internalizing and externalizing problems six weeks post-PICU discharge. Furthermore, younger age remained a significant factor related to psychosocial problems at six months post-PICU discharge, suggesting that age has an increasing impact over time. Our results contribute to the literature by demonstrating that younger PICU experience is linked to adverse psychological outcomes. These outcomes include more significant cognitive impairment [34], a reduced sense of control over health, and elevated symptoms of posttraumatic stress [35]. On the other hand, our results showed that older age is linked to greater adaptive functioning at six weeks and six months post-PICU discharge. Age has a moderating role in developing adaptive skills, as increases in chronological age coincide with gains in adaptive skill achievement [36]. Furthermore, a similar relationship between age and adaptive functioning has been noted in previous studies [37, 38].

Second, our results indicate that disease variables, the number of intervention procedures, and the severity of illness were shared risk factors for impaired psychosocial functioning at six weeks post-PICU discharge. These findings were consistent with previous studies that clarified the traumatic impact of illness severity and exposure to intervention procedures on children’s psychological outcomes [35, 39]. Moreover, in our study, length of stay was significantly associated with psychosocial problems up to six months post-PICU discharge. It is expected that an extended stay in the PICU may expose children to cumulative and repeated traumatic experiences, such as parental separation, troubled sleep, loud noises, and recurrent painful procedures, which in turn impact their psychosocial health. Furthermore, length of stay implies more extended recovery periods, which may, in turn, result in a vicious cycle of worsening psychological outcomes during the hospital stay and following discharge [40]. Notably, the length of stay was considered a consistent risk factor for adverse psychological outcomes in various studies [41, 42].

Third, our study identified parenting stress as a common theme associated with persistent psychosocial problems and adverse adaptive functioning up to six months post-PICU discharge, suggesting a potential role for psychological vulnerability. Parenting stress is a significant environmental risk factor that negatively impacts children’s behavioral development [43]. It can influence children’s learning and development, hindering skill acquisition and leading to behavioral problems [44]. Parents experiencing high levels of parenting stress may need help to meet their children’s needs [45]. They may be less effective in guiding their children through challenging situations [46]. According to the family systems model, higher stress perceived by parents may disrupt parent-child dyads, which, in turn, may lead to adverse child behavioral consequences [47]. Notably, parenting stress is a precursor and a result of child behavior problems. Therefore, parenting stress and child behavior issues appeared to influence each other, either escalating or de-escalating over time [48].

Finally, our study revealed that internalizing and externalizing problem behaviors were significantly related to adaptive functioning at six weeks and six months post-PICU discharge. These findings can reveal the influence of internalizing and externalizing problem behaviors on adaptive responses. For example, behavioral problems disrupt learning and strain relationships with teachers, peers, and parents [49]. Similarly, depression and anxiety hinder learning and limit access to opportunities for practicing adaptive skills [50]. It is increasingly recognized that poorer individual functioning and internalizing and externalizing problems co-occur [51, 52]. The effect may be bidirectional; for example, a child with externalizing problems may struggle academically or socially with their peers due to behavioral problems, and issues in one area can worsen problems in another [53].

### Limitations

The present study did not explore the link between PICU admission indications and psychosocial health or adaptive functioning. For example, a previous study revealed a relationship between congenital heart disease and extended adverse psychosocial outcomes [10]. Although the children in the PICU/ward group were matched by disease category, whether acute or chronic, a significant limitation of our findings is that we did not assess the prior functioning state of all patients at baseline. Evaluation of functional status is crucial at baseline and six months post-discharge as it may influence outcomes and link the findings to a PICU stay vs. an underlying chronic condition. Additionally, our study did not examine the prehospitalization psychological health of children or prior exposure to trauma as an indicator of psychological problems following PICU experience. Admission to a PICU is often unexpected, making it difficult to collect preadmission data and potentially leading to recall bias. However, one study showed that a prior history of posttraumatic stress was related to the emergence of acute stress following critical illness [24]. Another limitation is that we did not explore the bidirectional relationship between parental stress and children’s psychosocial health. Although our study captured the short-term and long-term impacts of PICU experience, it is worth extending the follow-up period to one to two years to identify the peak of psychological and adaptive behavioral difficulties and the period of stability and decline. Finally, the single-center design of our sample and the homogenous ethnic demography may restrict the generalizability of our findings.

### Implications

This study provides a broader view of the influence of PICU experience by studying children’s psychosocial and adaptive functioning after discharge for up to six months. At the time of discharge from the PICU, follow-up often focuses on medical aspects without considering broader impacts. Expanding the focus to include functional outcomes besides medical care is important. Our results revealed several PICU factors amenable to clinical intervention, such as the number of intervention procedures, illness severity, and length of stay. PICU staff should rationalize the necessity of repeating any intervention procedures and opting for the least painful procedure after careful evaluation without compromising the children’s care. Additionally, PICU staff should consider the advantages of nonpharmacological stress-reducing measures, such as improving sleep, providing distraction therapy [54], using music and massage [55], and explaining procedures to match the child’s developmental level to prevent the child from feeling confused or betrayed [5]. The PICU should provide a healthy environment to minimize the length of hospitalization. These include family inclusion, physical space, arts, and recreational activities, embracing child-friendly elements in the unit, and using ICU diaries to express emotions and communicate support [56].

Our study recognized that parenting stress was associated with worsened psychological outcomes in children post-PICU discharge during follow-up periods. Then, parents of children with experience in the PICU should undergo screening for psychological problems during and after their child’s discharge to identify potential areas for intervention. Existing evidence has shown that parents who receive psychological and emotional support, parental skills training, and family-centered nursing interventions demonstrate increased involvement in their children’s care and lower psychological problems post-discharge [57, 58].

The study findings revealed impaired psychosocial health and adaptive functioning in PICU survivors at six weeks and up to six months after discharge. These findings highlight the need for early screening of children during PICU hospitalization to promptly identify psychosocial problems and provide early psychotherapy interventions extending longitudinally after discharge at follow-up clinics. Teaching and fostering the acquisition of adaptive behavior should be the primary focus of any intervention, as it is also strongly associated with positive life outcomes [59]. Promoting adaptive functioning can help in managing self-control. Children with adaptive skills may face daily challenges with reduced aggression, disruptive behaviors, depression, and anxiety [50]. Hence, our findings strongly support the necessity for targeted intervention to enhance adaptive behavior based on children’s strengths and challenges. These function-based interventions require a multidisciplinary team of psychiatrists, psychologists, and social workers to address specific needs and provide personalized intervention plans. Adaptive behavior services provide a collaborative approach, including social skills training, problem-solving skills, flexibility in the face of change, goal-setting encouragement, and decision-making modeling. Regular assessment of adaptive functioning is essential for tracking improvements and adjusting intervention strategies.

## Conclusion

Our findings showed that PICU survivors continue to experience psychosocial problems and impaired adaptive functioning for up to six months after discharge. Our study identified several factors related to psychosocial health and adaptive functioning after discharge, such as parenting stress, the number of intervention procedures, and the severity of illness. In addition, internalizing and externalizing problems influenced adaptive functioning up to six months after PICU discharge. Understanding the clinical significance of these factors for psychological outcomes is essential for routine screening by healthcare personnel. Our research extends the current literature by examining adaptive functioning alongside psychosocial health. As a child has psychosocial symptoms, it is crucial to identify factors that influence adaptive functioning, particularly in clinical populations [60]. Improved adaptive skills will help enhance overall functioning in various community settings, making it a crucial focus [61]. Future longitudinal studies could explore how integrating early intervention programs focused on the adaptive functioning of PICU survivors during and after PICU discharge improves overall children’s functioning and psychosocial health development.

## Data Availability

All data generated or analyzed during this study are included in this published article or are available from the corresponding author upon reasonable request.

## Abbreviations

BASC-3: Behavioral Assessment System for Children, Third Edition
PSI-4-SF: Parenting Stress Index-Fourth Edition-Short Form
PICU: pediatric intensive care unit
PRISM: Pediatric Risk of Mortality-III
TISS-28: Therapeutic intervention scoring system

## References

[1] Rees G, Gledhill J, Garralda ME, Nadel S. Psychiatric outcome following paediatric intensive care unit (PICU) admission: A cohort study. Intensive Care Med. 2004;30(8):1607–14. 15112035 10.1007/s00134-004-2310-9

[2] Le Brocque RM, Dow BL, Mcmahon H, Crothers AL, Kenardy JA, Williams TJ, et al. The course of posttraumatic stress in children: examination of symptom trajectories and predictive factors following admission to pediatric intensive care. Pediatr Crit Care Med. 2020;21(7):E399–406. 32224826 10.1097/PCC.0000000000002316

[3] Baarslag MA, Jhingoer S, Ista E, Allegaert K, Tibboel D, van Dijk M. How often do we perform painful and stressful procedures in the paediatric intensive care unit? A prospective observational study. Aust Crit Care [Internet]. 2019;32(1):4–10. Available from: 10.1016/j.aucc.2018.04.003 10.1016/j.aucc.2018.04.003 29779912

[4] Davydow DS, Richardson LP, Zatzick DF, Katon WJ. Psychiatric morbidity in pediatric critical illness survivors. Arch Pediatr Adolesc Med. 2010;164(4):377–85. 20368492 10.1001/archpediatrics.2010.10 PMC2881634

[5] Rennick JE, Morin I, Kim D, Johnston CC, Dougherty G, Platt R. Identifying children at high risk for psychological sequelae after pediatric intensive care unit hospitalization. Pediatr Crit Care Med. 2004;5(4):358–63. 15215006 10.1097/01.pcc.0000128603.20501.0d

[6] Kyösti E, Ala-Kokko TI, Ohtonen P, Peltoniemi O, Ebeling H, Spalding M, et al. Strengths and difficulties questionnaire assessment of Long-Term psychological outcome in children after intensive care admission∗. Pediatr Crit Care Med. 2019;20(11):E496–502. 31274777 10.1097/PCC.0000000000002078

[7] Jacobs A, Dulfer K, Eveleens RD, Hordijk J, Van Cleemput H, Verlinden I, et al. Long-term developmental effect of withholding parenteral nutrition in paediatric intensive care units: a 4-year follow-up of the pepanic randomised controlled trial. Lancet Child Adolesc Heal. 2020;4(7):503–14. 10.1016/S2352-4642(20)30104-8 32562632

[8] Abend NS, Wagenman KL, Blake TP, Schultheis MT, Radcliffe J, Berg RA et al. Electrographic status epilepticus and neurobehavioral outcomes in critically ill children. Epilepsy Behav [Internet]. 2015;49:238–44. Available from: 10.1016/j.yebeh.2015.03.013 10.1016/j.yebeh.2015.03.013 PMC4536172 25908325

[9] Berger JT, Villalobos ME, Clark AE, Holubkov R, Pollack MM, Berg RA, et al. Cognitive development one year after infantile critical pertussis. Pediatr Crit Care Med. 2018;19(2):89–97. 29117060 10.1097/PCC.0000000000001367 PMC5796844

[10] Shevell AH, Sahakian SK, Nguyen Q, Fontela P, Rohlicek CMA. Associations between postoperative management in the critical care unit and adolescent developmental outcomes following cardiac surgery in infancy: an exploratory study. 2. Pediatr Crit Care Med. 2020;21(11):e1010–9. 32639471 10.1097/PCC.0000000000002398

[11] Buysse CMP, Vermunt LCAC, Raat H, Hazelzet JA, Hop WCJ, Utens EMWJ, et al. Surviving meningococcal septic shock in childhood: Long-term overall outcome and the effect on health-related quality of life. Crit Care. 2010;14(3):R124. 10.1186/cc9087. 10.1186/cc9087 PMC2911772 20587048

[12] Tassé MJ, Schalock RL, Balboni G, Bersani H, Borthwick-Duffy SA, Spreat S, et al. The construct of adaptive behavior: its conceptualization, measurement, and use in the field of intellectual disability. Am J Intellect Dev Disabil. 2012;117(4):291–303. 22809075 10.1352/1944-7558-117.4.291

[13] Reynolds CR, Kamphaus RW. (2015). BASC-3: Behavior Assessment System for Children, Third Edition. Bloomington, MN: NCS Pearson, Inc.

[14] Balboni G, Rebecchini G, Elisei S, Tassé MJ. Factors affecting the relationship between adaptive behavior and challenging behaviors in individuals with intellectual disability and co-occurring disorders. Res Dev Disabil. 2020;104:103718. 10.1016/j.ridd.2020.103718. 10.1016/j.ridd.2020.103718 32585440

[15] Biagas KV, Hinton VJ, Hasbani NR, Luckett PM, Wypij D, Nadkarni VM, et al. Long-Term neurobehavioral and quality of life outcomes of critically ill children after glycemic control. J Pediatr. 2020;218:57–e635. 31910992 10.1016/j.jpeds.2019.10.055 PMC7122648

[16] Moler FW, Silverstein FS, Holubkov R, Slomine BS, Christensen JR, Nadkarni VM, et al. Therapeutic hypothermia after Out-of-Hospital cardiac arrest in children. N Engl J Med. 2015;372(20):1898–908. 25913022 10.1056/NEJMoa1411480 PMC4470472

[17] Ebrahim S, Singh S, Hutchison JS, Kulkarni AV, Sananes R, Bowman KW, et al. Adaptive behavior, functional outcomes, and quality of life outcomes of children requiring urgent ICU admission*. Pediatr Crit Care Med. 2013;14(1):10–8. 23132399 10.1097/PCC.0b013e31825b64b3

[18] Ko MSM, Poh PF, Heng KYC, Sultana R, Murphy B, Ng RWL, et al. Assessment of Long-term psychological outcomes after pediatric intensive care unit admission: A systematic review and Meta-analysis. JAMA Pediatr. 2022;176(3):E215767. 35040918 10.1001/jamapediatrics.2021.5767 PMC8767488

[19] Abidin RR. Test Review: Parenting Stress Index, Fourth Edition (PSI-4). In: Lutz FL, editor. Parenting Stress Index. 4th ed. Lutz: PAR; 2012. 10.1177/0734282914556069.

[20] Fahmy SI, Nofal LM, Shehata SF, El Kady HM, Ibrahim HK. Updating indicators for scaling the socioeconomic level of families for health research. J Egypt Public Health Assoc. 2015;90(1):1–7. 25853538 10.1097/01.EPX.0000461924.05829.93

[21] Pollack MM, Patel KM, Ruttimann UE. PRISM III: an updated pediatric risk of mortality score. Crit Care Med. 1996;24(5):743–52. 8706448 10.1097/00003246-199605000-00004

[22] Miranda DR, De Rijk A, Schaufeli W. Simplified therapeutic intervention scoring system: the TISS-28 items - Results from a multicenter study. Crit Care Med. 1996;24(1 SUPPL):64–73. 8565541 10.1097/00003246-199601000-00012

[23] Dardas LA, Ahmad MM. Psychometric properties of the parenting stress index with parents of children with autistic disorder. J Intellect Disabil Res. 2014;58(6):560–71. 23701497 10.1111/jir.12053

[24] Nelson LP, Lachman SE, Goodman K, Gold JI. Admission psychosocial characteristics of critically ill children and acute stress. Pediatr Crit Care Med. 2021;22(2):194–203. 33156208 10.1097/PCC.0000000000002605 PMC8507146

[25] Timmermans M, Van Lier PAC, Koot HM. The role of stressful events in the development of behavioural and emotional problems from early childhood to late adolescence. Psychol Med. 2010;40(10):1659–68. 20056023 10.1017/S0033291709992091

[26] Caron C, Rutter M. Caron_Rutter_1991. 1991;32(7):1063–80. 10.1111/j.1469-7610.1991.tb00350.x 1787137

[27] Verstraete S, Verbruggen SC, Hordijk JA, Vanhorebeek I, Dulfer K, Güiza F, et al. Long-term developmental effects of withholding parenteral nutrition for 1 week in the paediatric intensive care unit: a 2-year follow-up of the pepanic international, randomised, controlled trial. Lancet Respir Med. 2019;7(2):141–53. 30224325 10.1016/S2213-2600(18)30334-5

[28] Slomine BS, Silverstein FS, Christensen JR, Page K, Holubkov R, Dean JM, et al. Neuropsychological outcomes of children 1 year after pediatric cardiac arrest: secondary analysis of 2 randomized clinical trials. JAMA Neurol. 2018;75(12):1502–10. 30242322 10.1001/jamaneurol.2018.2628 PMC6583192

[29] Hammen C. Generation of stress in the course of unipolar depression. J Abnorm Psychol. 1991;100(4):555–61. 1757669 10.1037//0021-843x.100.4.555

[30] Rnic K, Santee AC, Hoffmeister JA, Liu H, Chang KK, Chen RX, et al. The vicious cycle of psychopathology and stressful life events: A Meta-Analytic review testing the stress generation model. Psychol Bull. 2023;149:330–69. 37261747 10.1037/bul0000390

[31] Kira IA, Shuweikh H, Al-Huwailiah A, El-wakeel SA, Waheep NN, Ebada EE, et al. The direct and indirect impact of trauma types and cumulative stressors and traumas on executive functions. Appl Neuropsychol [Internet]. 2022;29(5):1078–94. Available from: 10.1080/23279095.2020.1848835. 10.1080/23279095.2020.1848835 33245250

[32] Lund JI, Toombs E, Radford A, Boles K, Mushquash C. Adverse childhood experiences and executive function difficulties in children: A systematic review. Child Abus Negl. 2020;106:104485. 10.1016/j.chiabu.2020. 10.1016/j.chiabu.2020.104485 32388225

[33] Cruz D, Lichten M, Berg K, George P. Developmental trauma: conceptual framework, associated risks and comorbidities, and evaluation and treatment. Front Psychiatry. 2022;13:800687. 10.3389/fpsyt.2022.800687. 10.3389/fpsyt.2022.800687 PMC9352895 35935425

[34] Bronner MB, Knoester H, Sol JJ, Bos AP, Heymans HSA, Grootenhuis MA. An explorative study on quality of life and psychological and cognitive function in pediatric survivors of septic shock. Pediatr Crit Care Med. 2009;10(6):636–42. 19581821 10.1097/PCC.0b013e3181ae5c1a

[35] Rennick JE, Johnston CC, Dougherty G, Platt R, Ritchie JA. Children’s psychological responses after critical illness and exposure to invasive technology. J Dev Behav Pediatr. 2002;23(3):133–44. 12055495 10.1097/00004703-200206000-00002

[36] Sparrow SS, Cicchetti DV, Balla DA. Vineland adaptive behavior scales. 2nd ed. Circle Pines, MN: American Guidance Service; 2005.

[37] Nazim A, Khalid R. Assessment of adaptive functioning of children with typical development attending primary schools in Lahore. Int J Soc Sci Educ Stud. 2018;4(4):124–35.

[38] Thomas SE, Kelly SJ, Mattson SN, Riley EP. Comparison of social abilities of children with fetal alcohol syndrome to those of children with similar IQ scores and normal controls. Alcohol Clin Exp Res. 1998;22(2):528–33. 9581664

[39] Muranjan MN, Birajdar SB, Shah HR, Sundaraman P, Tullu MS. Psychological consequences in pediatric intensive care unit survivors: the neglected outcome. Indian Pediatr. 2008;45(2):99–103. 18310787

[40] Ringdal M, Plos K, Lundberg D, Johansson L, Bergbom I. Outcome after injury: memories, health-related quality of life, anxiety, and symptoms of depression after intensive care. J Trauma - Inj Infect Crit Care. 2009;66(4):1226–33. 10.1097/TA.0b013e318181b8e3 19088550

[41] Sullivan E, Shelley J, Rainey E, Bennett M, Prajapati P, Powers MB et al. The association between posttraumatic stress symptoms, depression, and length of hospital stay following traumatic injury. Gen Hosp Psychiatry [Internet]. 2017;46:49–54. Available from: 10.1016/j.genhosppsych.2017.03.004. 10.1016/j.genhosppsych.2017.03.004 28622816

[42] Shears D, Nadel S, Gledhill J, Gordon F, Garralda ME. Psychiatric adjustment in the year after meningococcal disease in childhood. J Am Acad Child Adolesc Psychiatry [Internet]. 2007;46(1):76–82. Available from: 10.1097/01.chi.0000242234.83140.56. 10.1097/01.chi.0000242234.83140.56 17195732

[43] Mackler JS, Kelleher RT, Shanahan L, Calkins SD, Keane SP, O’Brien M. Parenting stress, parental reactions, and externalizing behavior from ages 4 to 10. J Marriage Fam. 2015;77(2):388–406. 26778852 10.1111/jomf.12163 PMC4712732

[44] Strauss K, Vicari S, Valeri G, D’Elia L, Arima S, Fava L. Parent inclusion in Early Intensive Behavioral Intervention: The influence of parental stress, parent treatment fidelity and parent-mediated generalization of behavior targets on child outcomes. Res Dev Disabil [Internet]. 2012;33(2):688–703. Available from: 10.1016/j.ridd.2011.11.008. 10.1016/j.ridd.2011.11.008 22188793

[45] Dubois-Comtois K, Moss E, Cyr C, Pascuzzo K. Behavior problems in middle childhood: the predictive role of maternal distress, child attachment, and mother-child interactions. J Abnorm Child Psychol. 2013;41(8):1311–24. 23748336 10.1007/s10802-013-9764-6

[46] Hustedt JT, Vu JA, Bargreen KN, Hallam RA, Han M. Early head start families’ experiences with stress: Understanding variations within a High-Risk, Low-Income sample. Infant Ment Health J. 2017;38(5):602–16. 28842928 10.1002/imhj.21667

[47] Bowen M. Family therapy in clinical practice. Northvale, NJ: Jason Aronson, Inc.; 1978.

[48] Neece CL, Green SA, Baker BL. Parenting stress and child behavior problems: A transactional relationship across time. Am J Intellect Dev Disabil. 2012;117(1):48–66. 22264112 10.1352/1944-7558-117.1.48 PMC4861150

[49] Masten AS, Roisman GI, Long JD, Burt KB, Obradović J, Riley JR, et al. Developmental cascades: linking academic achievement and externalizing and internalizing symptoms over 20 years. Dev Psychol. 2005;41(5):733–46. 16173871 10.1037/0012-1649.41.5.733

[50] Bornstein MH, Hahn CS, Suwalsky JTD. Developmental pathways among adaptive functioning and externalizing and internalizing behavioral problems: cascades from childhood into adolescence. Appl Dev Sci. 2013;17(2):76–87. 23585713 10.1080/10888691.2013.774875 PMC3622712

[51] Fanti KA, Henrich CC. Trajectories of pure and co-occurring internalizing and externalizing problems from age 2 to age 12: findings from the National Institute of child health and human development study of early child care. Dev Psychol. 2010;46(5):1159–75. 20822230 10.1037/a0020659

[52] Korhonen M, Luoma I, Salmelin RK, Helminen M, Kaltiala-Heino R, Tamminen T. The trajectories of child’s internalizing and externalizing problems, social competence and adolescent self-reported problems in a Finnish normal population sample. Sch Psychol Int. 2014;35(6):561–79.

[53] Korhonen M, Luoma I, Salmelin R, Siirtola A, Puura K. The trajectories of internalizing and externalizing problems from early childhood to adolescence and young adult outcome. J Child Adolesc Psychiatry [Internet]. 2018;2(3):7–12. Available from: https://www.pulsus.com/scholarly-articles/the-trajectories-of-internalizing-and-externalizing-problems-from-early-childhood-to-adolescence-and-young-adult-outcome.pdf.

[54] Kline WH, Turnbull A, Labruna VE, Haufler L, Devivio S, Ciminera P. Enhancing pain management in the PICU by teaching guided mental imagery: A quality-improvement project. J Pediatr Psychol. 2010;35(1):25–31. 19386770 10.1093/jpepsy/jsp030

[55] Küçük D, Bulut A, Yilmaz G. Impact of music therapy and hand massage in the pediatric intensive care unit on pain, fear and stress : Randomized controlled trial. Randomized controlled trial. 2023;71:95–103. 10.1016/j.pedn.2023.05.007 37230011

[56] Johansson M, Doctoral RN, Ingrid W. Family members ’ experiences with intensive care unit diaries when the patient does not survive. 2018;32(1):233–40. 10.1111/scs.12454. 10.1111/scs.12454 28524380

[57] Melnyk BM, Alpert-Gillis L, Feinstein NF, Crean HF, Johnson J, Fairbanks E, et al. Creating opportunities for parent empowerment: program effects on the mental health/coping outcomes of critically ill young children and their mothers. Pediatrics. 2004;113(6):e597–607. 10.1542/peds.113.6.e597. 10.1542/peds.113.6.e597 15173543

[58] Kasparian NA, Kan JM, Sood E, Wray J, Pincus HA, Newburger JW. Mental health care for parents of babies with congenital heart disease during intensive care unit admission: Systematic review and statement of best practice. Early Hum Dev. 2019;139:104837. 10.1016/j.earlhumdev.2019.104837. 10.1016/j.earlhumdev.2019.104837 31455569

[59] Tass MJ. Adaptive Behavior and Functional Life Skills Across the Lifespan: Conceptual Adaptive Behavior and Functional Life Skills Across the Lifespan: Conceptual and Measurement Issues. 2021.

[60] Jakupcak. Preval Psychol Correlates Complicated. 2007;20(3):251–62.

[61] Tassé MJ, Luckasson R, Schalock RL. The relation between intellectual functioning and adaptive behavior in the diagnosis of intellectual disability. Intellect Dev Disabil. 2016;54(6):381–90. 27893317 10.1352/1934-9556-54.6.381

